# The Comparative Effect on Satiety and Subsequent Energy Intake of Ingesting Sucrose or Isomaltulose Sweetened Trifle: A Randomized Crossover Trial

**DOI:** 10.3390/nu10101504

**Published:** 2018-10-15

**Authors:** Fiona E. Kendall, Olivia Marchand, Jillian J. Haszard, Bernard J. Venn

**Affiliations:** Department of Human Nutrition, University of Otago, P.O. Box 56, Dunedin 9054, New Zealand; fkendall831@gmail.com (F.E.K.); liv.marchand@gmail.com (O.M.); jill.haszard@otago.ac.nz (J.J.H.)

**Keywords:** satiety, sugars, sucrose, isomaltulose, glycemia

## Abstract

The effect that blood glucose concentration has on feelings of satiety is unclear. Our aims were to assess satiety and subsequent energy intake following the ingestion of trifle sweetened with sucrose or isomaltulose whilst measuring plasma glucose concentration to confirm glycemic differences between trifles. Seventy-seven healthy adults participated in a double-blind crossover trial where trifle sweetened with sucrose or isomaltulose was consumed on separate days with a two-week washout. Blood was sampled at the baseline, 1 and 2 h postprandially, and satiety assessed using visual analogue scales (VAS). Weighed diet records were taken on test days. A statistically significant difference in blood glucose concentration between trifles was found at 60 min following consumption, with the isomaltulose trifle having a 0.69 mmol/L (95% confidence interval: −1.07, −0.31) lower concentration when compared with the sucrose trifle. Mean satiety response by area-under-the-curve (AUC) was not significantly different between trifles. Mean (SD) appetite scores for the sucrose and isomaltulose trifles were 4493 (2393) and 4527 (2590) mm·min, respectively, with a between trifle difference of −9 (95% CI: −589, 572) mm·min. Mean (SD) energy intake for the remainder of the day following trifle consumption was 3894 kJ (1950 kJ) and 3530 kJ (1926 kJ) after the sucrose and isomaltulose trifles, respectively, and was not significantly different (*p* = 0.133). The differing glycemic response to trifle was not related to satiety or to subsequent energy intake.

## 1. Introduction

Overweight and obesity occur on a global scale and efforts are needed to counteract the problems of obesity-related diseases [1]. The World Health Organization estimated that 1.9 billion adults were overweight in 2016 [2]. A contributing factor to weight gain is likely to be the satiating property of foods [3]. Several factors have been variably associated with the satiating properties of food including protein content [4], fat content [5], fiber [6], and food volume [7]. Another factor suggested to regulate food intake is circulating blood glucose where it has been hypothesized that raised blood glucose concentrations promote satiety and low concentrations signal hunger [8]. A possible link between circulating blood glucose concentration and satiety has persisted with suggestions that diets producing low glycemic responses enhance weight control by promoting satiety [9,10]. A suggested mechanism is that slowly absorbed glucose interacts with nutrient receptors in the gut over an extended period, signaling prolonged satiety stimulus in the brain [11].

However, any effects of circulating blood glucose on satiety are unclear, as findings have been inconsistent [12]. Part of this inconsistency may be due to factors other than glycemic response that differ between test foods, for example, foods chosen on the basis of glycemic index (GI). Practical advice from the Glycemic Index Foundation is to exchange high for low GI foods [13]. If this advice is followed, there may be factors other than the glycemic response that differ between foods, for example, macronutrients [7] or fiber content [14]. 

The problem of attributing satiogenic effects to the glycemic response properties of foods selected on the basis of GI, independent of other properties of the food, has been reviewed [11]. The authors of the review found 14 studies by which to assess the effect of GI on satiety; in six of the studies, the fiber content of the lower GI foods was greater than that of the higher GI food; in another three studies, the low GI property of the test foods was achieved by adding extrinsic fiber; thus, the independent effect of the glycemic response per se on satiety has been difficult to assess [11]. It is possible to control for these food factors and in one such study, postprandial glycemia and satiety were found to differ in men consuming lunches containing different proportions of amylose to amylopectin in the starch fraction of otherwise comparable meals [15]. In a subsequent follow-up study by the same authors, despite lower glycemia after high were compared with the low amylose lunches, no differences in satiety were found [16]. 

Another strategy to manipulate glycemic responses by exchanging food ingredients is to use sugars with different GI. Isomaltulose (Palatinose™) is a non-cariogenic sugar found in trace amounts in honey [17] and in Japan, it has been commercially produced from sucrose and added to processed foods since the 1980s [18]. Isomaltulose is a structural isomer of sucrose, both disaccharides comprise one glucose and one fructose moiety but the glycosidic bond between the monosaccharides differs [19]. The different bonds result in isomaltulose being fully digested but at a slower rate than sucrose, creating a flattened blood glucose response curve following isomaltulose when compared with sucrose ingestion [20]. When groups of rats were sustained with these disaccharides over 24 h, it was found that food and energy intakes were lower in the animals fed isomaltulose when compared with the sucrose-fed group [21]. The effect on satiety of providing humans with foods containing these sugars has not been tested. 

The objective of this experiment was to compare the acute effect on satiety by incorporating a higher and a lower GI sugar as an ingredient into a solid food that could be consumed in practice. In order to test the hypothesis that feelings of satiety would be increased following the consumption of a food containing a lower GI ingredient, it was necessary to identify a food with a relatively high sugar content so that it would generate a difference in glycemic response between test foods. Trifle was chosen because each of its components (jelly, sponge, and custard) contain a considerable proportion of sugar. Hence, the current study was designed to measure glycemic responses and to test for short-term effects on satiety and subsequent energy intake by providing participants with trifle sweetened with either isomaltulose or sucrose. The main outcomes were satiety and subsequent energy intake, with plasma glucose concentration measured as confirmation of the effectiveness in generating glycemic differences between trifles.

## 2. Materials and Methods 

Participants were a convenience sample of students from the University of Otago. Inclusion criteria were students enrolled in a third year human nutrition course older than 18 years of age. Students were invited to participate in the study providing they had no food allergies to any of the trifle ingredients. Students were not obliged to take part and were given an information sheet and the opportunity to seek clarification of what the study involved. The University of Otago Human Ethics Committee approved the study (reference H17/011) and students signed a consent form. The study has been registered with the Australian and New Zealand Clinical Trials Registry ACTRN12618001137280.

### 2.1. Study Design

Seventy-seven young adults received a sucrose- or isomaltulose-sweetened trifle at lunchtime in a cross-over design randomized to the order in which they received the trifle. Participants attended two testing days on Fridays starting at 12 p.m. with a 2-week washout. Participants were stratified by the weeks when they could attend the test sessions to ensure equal distribution at each clinic for the order in which the trifles were consumed. 

The day before the first test day, each participant indicated to the investigators the type and amount of cereal that he or she wished to consume at breakfast the following morning and this was weighed and packaged in a sealable plastic bag for the participant to take home. Participants were instructed to eat all of the cereal on the morning of the test day at his or her usual breakfast time and then not to eat or drink (apart from water) until 12 p.m. Participants were provided with the same breakfast and asked to eat the breakfast at the same time on each of those days to ensure that appetite and energy intake prior to the lunchtime test sessions were consistent.

At 12 p.m., participants were seated in the testing facility and asked to consume the trifle within 20 min. No other food was consumed for the following 150 min. A staff member independent of the study used the random number generator in Excel (Microsoft, Redmond, Washington, WA, USA) to allocate the order in which each student received the sucrose- or isomaltulose-sweetened trifle. This staff member placed a colored sticker (red or green) onto the lid of the trifle container corresponding to sucrose or isomaltulose. The students, study investigators, and the biostatistician were blinded to trifle type with the colored code revealed after the completion of the statistical analysis. On the morning of the first test day, the participants’ heights were recorded to the nearest mm using a stadiometer (Holtain, Crymych, UK); and weight was measured to the nearest gram using calibrated electronic scales (Seca Deutschland, Hamburg, Germany). Body mass index was calculated as weight divided by height squared. Participants filled out a questionnaire regarding sex, age, and ethnicity.

### 2.2. Test Foods

The trifles were made in the metabolic kitchen of the Department of Human Nutrition at the University of Otago. The ingredients were: eggs, sugar (isomaltulose or sucrose in the form of castor sugar), plain flour, cornflour, baking powder, water, lemon juice, gelatin, full fat milk, and cream. Each serving weighed 446 g and contained 2600 kJ. The nutritional composition of the two trifles on a fresh weight basis were identical: protein 15.8 g; fat 18.6 g; available carbohydrate 98.2 g; total sugars 80.5 g (of which sucrose or isomaltulose 73.2 g); dietary fiber 0.6 g, and ash 2.1 g; with a moisture content of 70%.

### 2.3. Blood Testing

A 500 µL capillary blood sample was collected into a microcontainer containing potassium ethylenediaminetetraacetic acid (EDTA) (Becton, Dickinson and Company, Franklin Lakes, NJ, USA) using a contact-activated disposable lancet at the baseline and at 1 and 2 h following consumption of the trifles. The tubes were centrifuged for 10 min at 2000× *g* within 20 min of blood collection and the plasma was extracted and stored at −80 °C until analysis. Plasma glucose concentration was measured using the glucose hexokinase method on a Cobas c311 auto analyzer (Roche Diagnostics, Indianapolis, IN, USA). Coefficients of variation for Roche control sera Precinorm U plus (nominal 4 mmol/L) and Precipath U plus (nominal 13 mmol/L) were 1.25% and 0.67%, respectively. 

### 2.4. Satiety and Dietary Recording

Feelings of satiety were assessed using 100 mm Visual Analogue Scales (VAS) using methodology validated by Flint and colleagues [22]. Four questions were asked with anchoring statements as given in parentheses “How hungry do you feel?” (I am not hungry at all/I have never been more hungry); “How satisfied do you feel?” (I am completely empty/I cannot eat another bite); “How full do you feel?” (Not at all full/Totally full); and “How much do you think you can eat?” (Nothing at all/A lot). The set of four questions were asked at the baseline and at 30, 60, 90, 120, and 150 min after eating the trifles. Responses to each question at each timepoint were marked on a 100 mm line and the sheets removed. Each of the four satiety questions were analyzed for each person using area under the curve over 150 min (AUC). In addition, a composite appetite score was generated by taking the average AUC of the four questions at each time point. Cronbach’s alphas were calculated for the overall appetite scale at each time point.

Training was given to participants in the use of the Model 3010 Salter electronic kitchen scales reading to 1 g (Salter Housewares, Tonbridge, UK). Participants took the scales home and weighed and recorded all food and beverages consumed throughout the day from waking on the morning of each test day through to midnight. The dietary data were entered into a University of Otago dietary analysis program that uses the New Zealand Food Composition database as the source of nutrient information [23].

### 2.5. Statistical Analysis

A sample of 60 was required to detect a difference of 0.5 standard deviations for all outcomes in standardized form with 90% power and α = 0.01. Seventy-seven participants were recruited as a convenience sample, which allowed for some dropout. Random effects regression analysis was used to test for between-treatment differences in plasma glucose at the 60 and 120 min timepoints and for AUC satiety responses with participant id as a random effect and adjusted for randomized order and baseline satiety. Analysis was also undertaken for standardized AUC and to estimate differences in subsequent energy intake. A *p*-value < 0.05 was considered statistically significant. Stata 15.1 (StataCorp, College Station, TX, USA) was used to analyze the VAS satiety data.

## 3. Results

**Figure 1:**
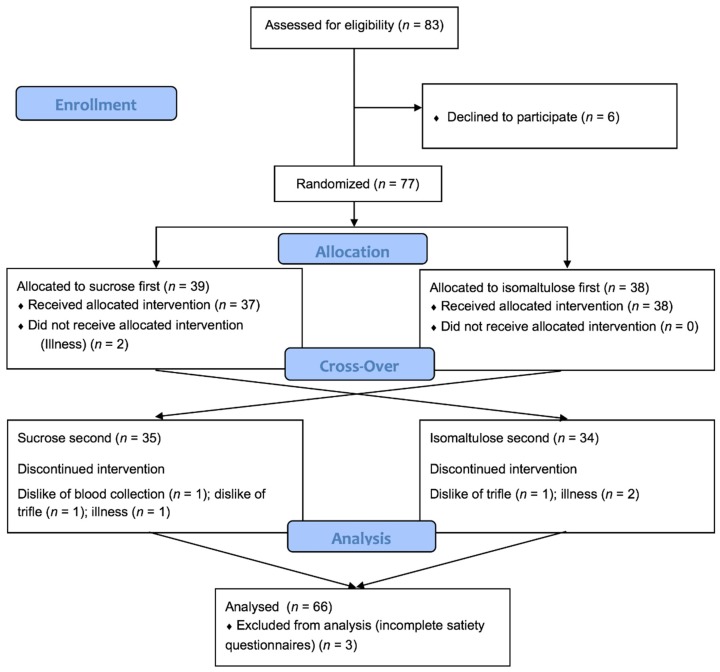
CONSORT diagram showing the flow of participants through the study.

Seventy-seven participants were randomized to order and complete blood and satiety data were available for 66 people. A diagram of participant flow through the study is given in Figure 1. 

Anthropometric and demographic characteristics of the sample are given in Table 1.

Participants were mainly young female adults of European descent, with Asian and Māori ethnicities combined constituting 30% of the sample.

### 3.1. Blood Glucose

The mean (SD) plasma glucose concentrations at the baseline were 5.2 (0.7) and 5.1 (0.7) mmol/L for the sucrose and isomaltulose-sweetened trifles, respectively, and these concentrations were not different (*p* = 0.253). The mean blood glucose concentration data sampled at 60 and 120 min and comparisons between treatments at 60 and 120 min are given in Table 2. 

Blood glucose rose at 60 min then declined at 120 min, though remained above the baseline for both trifles. A statistically significant difference between trifles was observed at 60 min following consumption.

### 3.2. Satiety

AUC was used to measure appetite response across the testing time period on both days, spanning the baseline (prior to trifle ingestion) to 150 min postprandial. This enabled six VAS questionnaires to be completed by each participant on a given testing day. There were no significant differences for mean difference between the isomaltulose- and sucrose-sweetened trifle or in mean AUC for each satiety question across all time points following the consumption of the trifles (Table 3).

A meaningful effect was ruled out as the mean difference and 95% CI were all under 0.3 standard deviations. Therefore, it is unlikely that there is a real difference in satiety between the trifles. Sixty-six participants recorded subsequent energy intake for the rest of the day after the trial; mean (SD) energy intake after the sucrose trifle was 3894 kJ (1950 kJ), and after the isomaltulose trifle it was 3530 kJ (1926 kJ). Energy intake for the remainder of the day after consuming the trifle did not differ significantly between treatments with a mean difference (sucrose arm-isomaltulose arm) of 364 kJ (95% CI: −110 kJ, 838 kJ), *p* = 0.133.

## 4. Discussion

In the present study, differences in postprandial glycemia were found between trifles, but there were no significant differences in the participants feelings of satiety or in their subsequent energy intake. These findings are consistent with other work. When comparing satiety among 38 foods, food volume or energy density were found to be the strongest predictors of satiety index scores, with satiety index defined as area under the 120 min satiety curve (AUC) of the test food divided by the AUC of white bread [24]. Using the same index, the portion sizes of seven isocaloric breads were predictors of satiety and subsequent energy intake with no significant relationship found between glycemic response and satiety [7]. We controlled for both volume and energy density as the trifles were identical in these factors. Thus, there is consistency that volume or energy density of foods are predictive of satiety, whereas differences in glycemia, at least of the magnitude attained in these studies, is not.

In contrast, differences in some appetite measures have been found from studies where comparison treatments have been designed using food choices based on GI. In one such study, food was requested approximately three-quarters of an hour earlier after the high GI meal when compared with the low GI meals, although there was no difference in subsequent energy intake [25]. In a study in which shepherd’s pie contained either low GI beans or high GI potato puree, feelings of hunger were delayed and stomach fullness was greater four hours after eating the bean when compared with the potato meal [26]. The authors of that study were unable to exclude the possibility that factors other than glycemic responses were influential over satiety, as the nutrient compositions of the meals differed [26]. In a longer-term crossover study conducted over 28 days using low and high GI foods, the mean hunger rating of 80 participants over the study period was not different between diets, but people reported feeling fuller while eating the low GI diet [14]. 

The outcomes of these studies are variable both within and among studies, but in each study there was some indication that low, as opposed to high GI foods, resulted in some greater measure of satiety. However, whether any of the differences found were due to glycemic responses is uncertain. The fiber content of foods has been found in some studies to affect satiety [6]. In the study by Chang et al., the low GI diets contained 55 g/day fiber, considerably more than the 28 g/day in the high GI diets [14]. In the study by Leathwood and colleagues, the shepherd’s pie containing bean puree had more protein, less carbohydrate, and more fiber (13 vs. 6 g) than the potato meal [26]. The fiber content was not reported by Ball et al., but the low GI foods were products designed to contain relatively high fiber contents (USANA, Salt Lake City, UT, USA) while the high GI products comprising a maltodextrin based beverage and an Ensure bar (Ross Products Division, Abbott Laboratories, Columbus, OH, USA) were likely to have contained less fiber [25]. Thus, because different foods were used to generate glycemic differences between treatment arms, it is possible that factors other than glycemic responses may have contributed to, or have been the cause of, differences in satiety.

Controlling for factors such as fiber, energy, and macronutrient content is possible with the use of beverages sweetened with sugars with different glycemic-inducing characteristics. Beverages sweetened with glucose (G) and fructose (F) mixtures of G80:F20 (high glycemic) and G20:F80 (low glycemic) ratios were given to 12 and 19 people (two experiments), resulting in no difference in the ratings of appetite but a lower subsequent energy intake 80 min after drinking the high-compared with the low-glycemic beverage [27]. In another trial involving 15 adolescents, subsequent food intake was lower after a glucose beverage compared with a sucralose control; and appetite ratings were higher after ingesting a glucose beverage compared with a high-fructose corn syrup beverage [28]. These data are suggestive that glucose has a satiating effect, potentially via its glycemic-raising capacity in accordance with the glucostatic theory where an elevated blood glucose concentration is hypothesized to induce appetite dampening [8]. However, fructose undergoes different metabolic processes to glucose and therefore it is possible that differences in satiety may result from differences in the metabolism of the two sugars, for example, via cerebral blood flow acting on appetite signals [29].

A means of isolating the glycemic effects of sugars on satiety is the use of isomaltulose and sucrose as the comparison treatments as these two sugars have identical monosaccharide constituents. It has been found that rats provided with sucrose or isomaltulose ingested more energy over 24 h when exposed to sucrose compared with isomaltulose [21]. However, these were extreme diets in that the intakes were 100% of either sugar in an animal model. In humans, using a practical approach, the trifles our participants consumed generated differences in glycemia, but resulted in no difference in the immediate ratings of satiety or in subsequent energy intake throughout the day. A limitation of our work was the infrequent sampling of blood glucose, at the baseline, one and two hours later. The infrequency was to avoid participant anxiety at having multiple fingerpricks taken during the time when subjective feelings of satiety were being collected. Nevertheless, we were able to confirm a significant difference in glycemic response at the one-hour timepoint. Generalizability may also be limiting as our participants were young, healthy, and predominantly female. A difference in feelings of fullness over time following ad libitum consumption of yogurt between adolescent and elderly participants has been found [30],however, it is unknown how age would effect change in satiety when comparing between two test foods.

A major strength of the study was the use of isomaltulose and sucrose as the sweeteners that allowed for double-blinding and for the control of many factors associated with satiety including volume of food, macronutrients, fiber, and energy content. The study also had a strong design, being a crossover, participants were randomized to treatment order, and it was adequately powered with a relatively large sample. A limitation was the inclusion of these sugars into trifle that limited the glycemic difference between treatments. A maximum difference of around 1.5 mmol/L in blood glucose concentration between sucrose and isomaltulose has been found when participants ingested 50 g solutions of these beverages [20] whereas the difference between trifles was 0.69 mmol/L using 73 g of the sugars. The reason for the diminished glycemic difference could be that the infrequency of sampling missed the time of maximum separation, or could be due to the inclusion of fat and protein in the trifles. Co-ingestion of fat and carbohydrate lessens the glycemic response when compared with carbohydrate alone [31] and protein stimulates insulin, thereby encouraging glucose disposal out of circulation [32]. It is possible that greater differences in glycemia could be related to satiety and this could be tested by feeding sucrose or isomaltulose beverages without the addition of fat and protein; or by increasing still further the amount of these sugars incorporated into test foods. Generalizability is also limiting as our sample was predominantly young, healthy females. Testing for the effects of these sugars on the satiety of other age groups and in people with impaired glucose tolerance would be informative.

## 5. Conclusions

In conclusion, sucrose and isomaltulose contain identical glucose and fructose molecules but differ in the glycosidic bond joining the monosaccharides, resulting in the slower digestion of isomaltulose when compared with sucrose. The slower rate of digestion of isomaltulose compared with sucrose generated a glycemic difference between the two trifles at lunchtime, but this glycemic difference did not result in differences in feelings of satiety or in subsequent food intake over the remainder of the day. These data are novel and will hopefully lead to other investigators testing the satiating properties of these sugars amongst a wider demographic.

## Figures and Tables

**Table 1:** Baseline demographics and characteristics (*n* = 77).

Characteristic	Mean (SD) or *n* (%)
Height (m)	1.7 (0.1)
Weight (kg)	66.4 (13.5)
Body Mass Index (kg/m^2^)	23.7 (3.6)
Sex n (Female/Male)	59 F/18 M
Age (year)	21.9 (5.6)
Ethnicity *n* (%)	
New Zealand European	49 (65%)
Asian	17 (23%)
Māori	5 (7%)
Other	4 (5%)

**Table 2:** Blood glucose concentrations (mmol/L) and difference between trifles (*n* = 66).

Time (min)	Sucrose Mean (SD)	Isomaltulose Mean (SD)	Isomaltulose-Sucrose Mean Difference (95% Confidence Interval) ^1^	*p*
60	7.3 (1.7)	6.6 (1.1)	−0.69 (−1.07, −0.31)	<0.001
120	5.9 (0.9)	6.1 (0.9)	0.18 (−0.10, 0.45)	0.215

^1^ Random effects regression analysis adjusted for baseline and order of treatment.

**Table 3:** Subjective satiety area under the curve (AUC) using visual analogue scales over 150 min (*n* = 66).

VAS Question	Sucrose Mean (SD) mm·min	Isomaltulose Mean (SD) mm·min	Mean Difference (95% CI) mm·min	Mean Standardized Difference (95% CI)
How hungry do you feel?	3628 (2457)	3697 (2454)	37 (−616, −691)	0.02 (−0.25, 0.28)
How satisfied do you feel?	4928 (2506)	4886 (2667)	−97 (−717, −523)	0.04 (−0.28, 0.20)
How full do you feel?	4768 (2668)	4899 (2859)	23 (−673, −718)	0.01 (−0.24, 0.26)
How much do you think you can eat?	4718 (2777)	4729 (2979)	9 (−600, −617)	0.00 (−0.21, 0.22)
Overall appetite score ^a^	4493 (2393)	4527 (2590)	−9 (−589, −572)	0.00 (−0.24, 0.23)

^a^ The overall appetite score was an average of the AUC values of the four satiety questions. The questions were highly correlated with internal reliability (Cronbach’s alpha) of 0.86–0.94 at each time point. There were no significance between-trifle differences for any of the questions. VAS: visual analogue scales.
